# Gadoxetate Acid-Enhanced MR Imaging for HCC: A Review for Clinicians

**DOI:** 10.4061/2011/489342

**Published:** 2011-07-13

**Authors:** Jendana Chanyaputhipong, Su-Chong Albert Low, Pierce K. H. Chow

**Affiliations:** ^1^Department of General Surgery, Singapore General Hospital, 169608, Singapore; ^2^Department of Diagnostic Radiology, Singapore General Hospital, Outram Road, 169608, Singapore; ^3^Duke-NUS Graduate Medical School, 169857, Singapore

## Abstract

Hepatocellular carcinoma (HCC) is increasingly being detected at an earlier stage, owing to the screening programs and regular imaging follow-up in high-risk populations. Small HCCs still pose diagnostic challenges on imaging due to decreased sensitivity and increased frequency of atypical features. Differentiating early HCC from premalignant or benign nodules is important as management differs and has implications on both the quality of life and the overall survival for the patients. Gadoxetate acid (Gd-EOB-DTPA, Primovist^®^, Bayer Schering Pharma) is a relatively new, safe and well-tolerated liver-specific contrast agent for magnetic resonance (MR) imaging of the liver that has combined perfusion- and hepatocyte-specific properties, allowing for the acquisition of both dynamic and hepatobiliary phase images. Its high biliary uptake and excretion improves lesion detection and characterization by increasing liver-to-lesion conspicuity in the added hepatobiliary phase imaging. To date, gadoxetate acid-enhanced MRI has been mostly shown to be superior to unenhanced MRI, computed tomography, and other types of contrast agents in the detection and characterization of liver lesions. This review article focuses on the evolving role of gadoxetate acid in the characterization of HCC, differentiating it from other mimickers of HCC.

## 1. Brief Overview of HCC

Hepatocellular carcinoma (HCC) is the fifth most common malignant neoplasm and the third most common cause of cancer-related death worldwide [1]. There has been a reported 41% increase in mortality from HCC over the last 2 decades [2], and HCC continues to be a major health concern. Many studies have shown that patients with early-stage HCC, as defined by the Milan criteria [3], treated either by resection [4, 5] or transplantation [3], do significantly better than those with advanced disease [6], with 5-year overall survival rate approximating 40–70% [6, 7] in such cases. The presence of microvascular invasion—an independent poor prognostic factor regardless of treatment—is more probable in larger tumors [8–10]. Thus, the detection and accurate characterization of early focal liver lesion in normal or cirrhotic livers is crucial so that appropriate treatment can be instituted [11–13].

## 2. The Evolution in Magnetic Resonance Imaging (MRI) of the Liver

MRI has become an established modality for the assessment of various types of focal liver lesions [14–18]. Nevertheless, up to 60% of small malignant nodules, particularly those less than 1 cm size in the background of cirrhotic liver, are missed at MRI [19]. Continued improvement in the MR sequences and hardware [20, 21], as well as the advent of liver-specific contrast agents [22, 23], which are only available for MRI, have led to the improved diagnostic performance of MRI. The broad arsenal of MR sequences and multiphasic postcontrast imaging provide comprehensive information on the liver lesion by elucidating different signal intensities that reflect the inherent properties of the lesion's composition, as well as blood flow dynamics, which gives each lesion type different MR characteristic appearances.

## 3. Liver-Specific Contrast Agents for MRI

### 3.1. An Overview

To increase the sensitivity and specificity of MRI in the detection and characterization of focal liver lesions and overcome some of the existing limitations of extracellular fluid (ECF) agents, which include suboptimal differentiation between benign and malignant liver lesions due to the contrast agents' non-specific nature and nephrotoxicity (nephrogenic systemic fibrosis) that can result with use of high doses of gadolinium contrasts [24], liver-specific contrast agents emerged. Currently, two major classes of liver-specific contrast agents exist: (1) hepatocyte-specific, or hepatobiliary, agents and (2) reticuloendothelial cell-specific, or nanoparticulate, agents. They are considered “liver-specific” as they all cause significant liver signal changes after intravenous administration, with resultant increased liver-to-lesion conspicuity. The first group of contrast agents, as the name implies, targets the functioning hepatocytes with varying degree of contrast uptake into them with subsequent biliary excretion. This is possible because of the addition of a lipophilic moiety to the gadolinium chelates [25]. Currently available contrast agents of this type include gadoxetate acid (Gd-EOB-DTPA or gadolinium ethoxybenzyl diethylene-triamine pentaacetic acid, Primovist^®^, Eovist^®^ in the USA, Bayer Schering Pharma, Berlin, Germany) and gadobenate dimeglumine (Gd-BOPTA, Multihance^®^, Bracco, SpA, Milan, Italy), both of which are gadolinium-based. Manganese-based paramagnetic agent, mangafodipir trisodium (Mn-dipyridoxyl 5'phosphate, Teslascan^®^, GE Healthcare, Oslo, Norway), was another contrast agent belonging to this group; however, it has been removed for use in the United States [26] and will not be further discussed here. 

The second group of contrast agents target the Kupffer cells of the reticuloendothelial system, where phagocytosis of contrast agents occur and, by the effects of iron ions, liver signal intensity decreases giving rise to a “black” liver [27], instead of “white” liver seen with hepatocyte-specific contrast agents.

### 3.2. Hepatobiliary Agents

**Gadoxetate Acid**

Gadoxetate acid is a gadolinium-based, paramagnetic, liver-specific MR contrast agent with combined perfusion- and hepatocyte-selective properties that is primarily developed for imaging of the liver to improve lesion detection and characterization. It has been found in preclinical studies to be safe and well tolerated with no major side effects [25, 28–30].

Several unique properties deserve mention. Upon intravenous administration of gadoxetate acid, it rapidly distributes itself in the vascular-interstitial compartment, enhancing the blood pool, providing acquisition of dynamic phase images that allows for lesion characterization based on perfusion. Approximately 50% of the injected dose of gadoxetate acid is then selectively taken up by the functioning hepatocytes and subsequently excreted into bile, allowing for the acquisition of the delayed, hepatocyte-specific phase that is optimal at 20 min post injection. This phase further improves diagnostic performance by increasing liver-to-lesion contrast, where lesions with absent or dysfunctional hepatocytes appear dark against the background white liver. Because of such high specificity for hepatocytes, the recommended dose of gadolinium is 4-fold less than the ECF agents [25, 29, 30].

The cellular mechanism underlying this high percentage of contrast volume uptake can be explained by the enhanced lipophilic property of gadoxetate acid due to the presence of EOB moiety that is linked to the gadolinium complex. Passive diffusion of contrast agent occurs via transporter molecules, organic anion transporting polypeptide 1 (OATP1), that are present on the basolateral membrane of the normal hepatocytes [31–33].

Following a relatively high hepatocyte uptake, studies have shown that gadoxetate acid is cleared in equal quantities via bile (50%) and urine (50%). At molecular level, its excretion into bile is as a result of another type of transporter molecule present at the canalicular membrane of the cell called multidrug resistance protein 2 (MRP2) [31–33]. In the event that one of these elimination pathways is impaired, the other elimination pathway compensates, according to animal studies [34, 35]. This theoretically allows patients with either renal or liver impairment to safely undergo examination by gadoxetate acid-enhanced MRI, although to date, there is no human studies to confirm this.

Gadoxetate acid is also highly water-soluble and thus is bolus-injectable [29, 30]. Previous non-gadolinium liver-specific contrast agents did not allow for a single examination of both the vascular- and the liver-specific phase to be performed after a single injection in a reasonable time-frame. However, gadoxetate acid-enhanced MRI is injected as a bolus and allows for the acquisition of the delayed (hepatocyte-specific) phase at 20 minutes post injection via the mechanism described above, with a total examination time possible in 35 min.

The diagnostic performance of gadoxetate acid-enhanced MRI versus other forms of imaging or other contrast agents for MRI will be discussed in a separate section below.

**Gadobenate Dimeglumine**

Gadobenate dimeglumine (Gd-BOPTA; Multihance^®^, Bracco SpA, Milan, Italy), like gadoxetate acid, is a gadolinium-based, dual-acting (with combined extracellular and liver-specific properties) contrast agent, that provides two-level information of a suspected lesion: its vascularity (from the dynamic phase imaging) and its cellularity (from the hepatobiliary phase imaging). It has been shown to be safe and well-tolerated in preliminary studies [36–38].

One of the main differences between the two contrast agents (see Table 1) is the degree of hepatocyte uptake. With gadobenate dimeglumine, only 2–4% (as compared to 50% of gadoxetate acid) is taken up by functioning hepatocytes; it is predominantly (96%) cleared by the kidneys [37]. This has several implications: (1) theoretically, the higher proportion of contrast elimination via the kidneys means patients with significant renal impairment should not be advised to undergo MR studies with this contrast; (2) acquisition time of the hepatocyte-specific phase occurs later than that of gadoxetate acid (40 min versus 20 min), (3) recommended dosage of contrast volume is different (higher with gadobenate dimeglumine) [36–39]. Despite the differences in the degree of hepatocyte uptake and the time course of liver enhancement, it has been found that both agents, during their maximum enhancement, provide comparable enhancement of the liver parenchyma [40]. For gadobenate dimeglumine, this is achieved because OATP phosphorylation—occurs when the agent is taken up into the hepatocytes—causes changes in MRP2 location and expression, preventing the exit of contrast material into bile [41, 42].

Several studies have demonstrated superior diagnostic performance of gadobenate dimeglumine-enhanced MRI in the detection and characterization of benign and malignant liver nodules over non-specific extracellular agents and ferumoxides [43–48]. In the detection of HCC, Choi et al. [49] reported a sensitivity of 80–85% and a positive predictive value of 65-66%.

### 3.3. Reticuloendothelial Cell-Specific Agents

Superparamagnetic iron oxide (SPIO) is another class of liver-specific contrast agents for MR imaging of the liver. Ferucarbotran (Resovist^®^; Bayer Schering, Berlin, Germany), a commonly used SPIO, works by targeting the Kupffer cells of the reticuloendothelial system (RES), which are present in various organs, including the liver, spleen, and bone marrow [50]. It is also administered intravenously as a bolus [51]. Unlike gadoxetate acid that can evaluate a liver lesion by its function and vascularity, SPIO can only evaluate a lesion functionally. 

Generally, malignant lesions (HCC) are presumed to lack phagocytic activity and thus appear hyperintense with respect to the hypointense liver parenchyma on SPIO-enhanced MRI [27, 52]. This differs from findings of hepatocyte-specific contrast-enhanced MRI, where most HCC nodules appear hypointense with respect to the hyperintense liver parenchyma in the hepatobiliary phase. However, it is important to note that up to 60% of well-differentiated HCCs are not hyperintense on ferucarbotran-enhanced MRI possibly due to the fact that early HCCs may retain normal Kupffer cell function and counts [53–55].

Table 2 summarizes the major differences between the two types of liver-specific contrast agents.

## 4. Gadoxetate Acid for Detection and Characterization of HCC

The liver parenchyma enhances strongly in the hepatocyte phase on T1-weighted images, starting at 10–20 min after the intravenous injection of contrast. This forms the background against which various types of nodules, which do or do not contain functioning hepatocytes, stand out. Nodules that do not contain normal functioning hepatocytes, such as most HCC or liver metastases, lack contrast uptake and are usually depicted as low-intensity (hypointense) lesions. On the other hand, nodules that do contain (varying degrees of) functioning hepatocytes, such as regenerative nodules of focal nodular hyperplasia (FNH), appear enhanced, either to a similar or higher degree to the surrounding liver parenchyma.

**HCC**

Using AASLD criteria [56], HCC can be diagnosed noninvaively in at-risk patients with contrast-enhanced imaging, typically showing arterial phase enhancement and venous or delayed phase washout on CT or MRI [57, 58]. The presence of fat or late enhancing pseudocapsule are supportive features. Complementary features on MRI include mild-moderate hyperintensity on T2-weighted images and restricted diffusion on diffusion-weighted imaging (DWI) sequences. With the recent international consensus recognition of the early HCC nodule as a pathologic entity, their imaging correlates are also being increasingly recognized at hepatobiliary phase imaging as the decreased expression of anion transporters may predate the development of overt hypervascularity. At conventional dynamic contrast-enhanced imaging, a significant proportion of these early HCCs will not show typical diagnostic arterial phase hyperenhancement and would be potentially misdiagnosed as benign lesions, such as regenerative or dysplastic nodules. At hepatobiliary phase imaging post gadoxetate acid administration, 3 patterns of HCC have been described, depending on whether they express transporter molecules OATP1 [31] on their membranes: (1) typically, as arterial hypervascularized lesion and washout on a 3-min late phase MRI and hypointense lesion at 10–20-min hepatocyte phase because most HCCs do not contain functioning hepatocytes and hence >80% of HCCs appear hypointense in relation to the surrounding enhanced liver parenchyma [59, 60]; (2) as isointense or hyperintense lesions at 10–20-min hepatocyte phase because some moderate or well-differentiated HCCs may overexpress anion transporters OATP1 resulting in uptake of contrast agent in 10–20% of cases [59, 60]; (3) occasionally in approximately 10% of HCCs especially small lesions may present as hypointense lesions on hepatocyte phase imaging without accompanying arterial hypervascularization or T2-weighted or DWI hyperintensity [61].

The following underlying cellular mechanism explains the above phenomena. In a normal liver, after intravenous administration, gadoxetate acid first reaches the extracellular space (the vasculature). It then enters the normal functioning hepatocytes via transporter molecule organic anion transporting peptides (OATPs) that are located in the hepatocyte's basolateral membrane. The contrast agents then exits the hepatocytes into bile (in 50% of injected contrast volume) through another transporter molecule located on the canalicular membrane, the multidrug resistance protein 2 (MRP2) [31–33]. In cirrhotics, these two transporter molecule expressions undergo modifications. It has been established that the presence of OATPs determines the uptake of gadoxetate acid in hepatocellular carcinoma [62]. In 2010, Tsuboyama et al. [63] found that when OATPs are present in HCC, the expression and location of MRP2 is the one ultimately responsible for the cellular accumulation or lack of it. If the MRP2 are present on the normal canalicular membrane, the contrast material will exit into bile and that HCC nodule will appear hypointense. Correspondingly, Tsuda and Matsui [64] found that the presence of liver cirrhosis upregulates MRP2, which promotes the elimination of gadoxetate acid. Thus, although some HCCs may contain OATPs, most still appear hypointense relative to the liver enhancement. On the contrary, if MRP2 is situated in the pseudoglands, the contrast agent will not be able to exit into bile, and its accumulation in the HCC lesion causes it to appear hyperintense [63]. A similar report regarding above findings with use of gadobenate dimeglumine has been described by Planchamp C and team in his animal study [41, 42].

**Figure 1:**
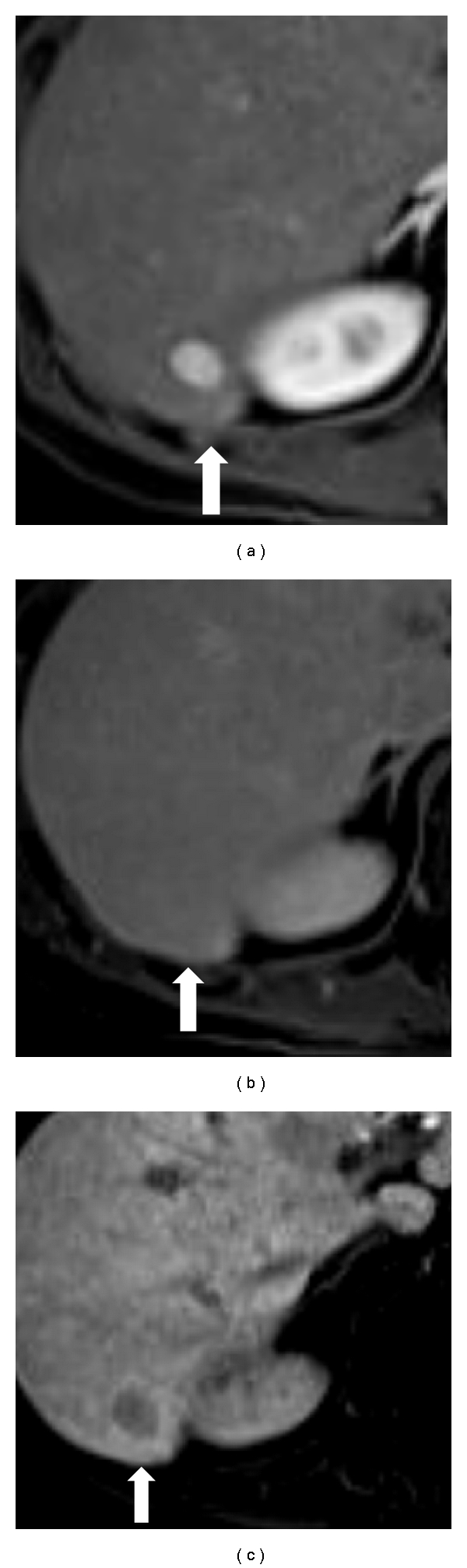
(a) Gadoxetate acid-enhanced MRI in the arterial phase in a 51-year-old male with alcoholic liver cirrhosis showing a hyperenhancing nodule in the liver segment 6. (b) Equilibrium phase imaging shows isointense appearance with no hypointense washout. The diagnosis of HCC is therefore not confirmed in the dynamic vascular phases. (c) Hepatobiliary phase imaging at 20 minutes after injection shows a hypointense nodule against the background of enhancing liver parenchyma, implying lack of lesional uptake. This additional information allowed more confident diagnosis of HCC. Final histopathology was a well-differentiated Edmondson-Steiner grade I HCC.

**Figure 2:**
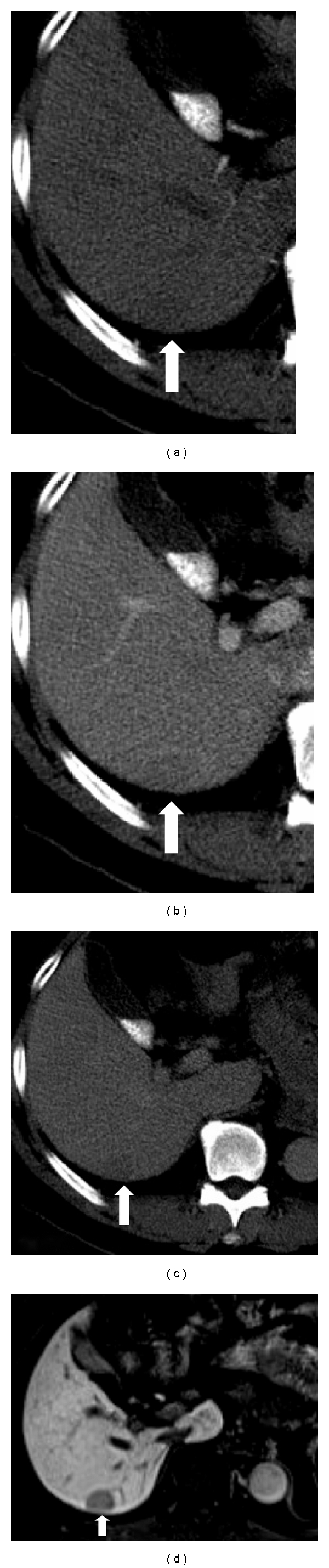
(a–c) Contrast-enhanced CT in the arterial, venous and equilibrium phases of a 75-year-old male Hepatitis B virus carrier showing an indeterminate slightly hypodense nonhypervascular nodule in the liver segment 6. (d) Gadoxetate acid-enhanced MRI in the hepatobiliary phase 20 minutes after injection showing a hypointense nodule against the background of enhancing liver parenchyma, implying lack of lesional uptake, suspicious for HCC or high-grade dysplastic nodule. Final surgical histopathology was a well-differentiated Edmondson-Steiner grade I HCC.

**Figure 3:**
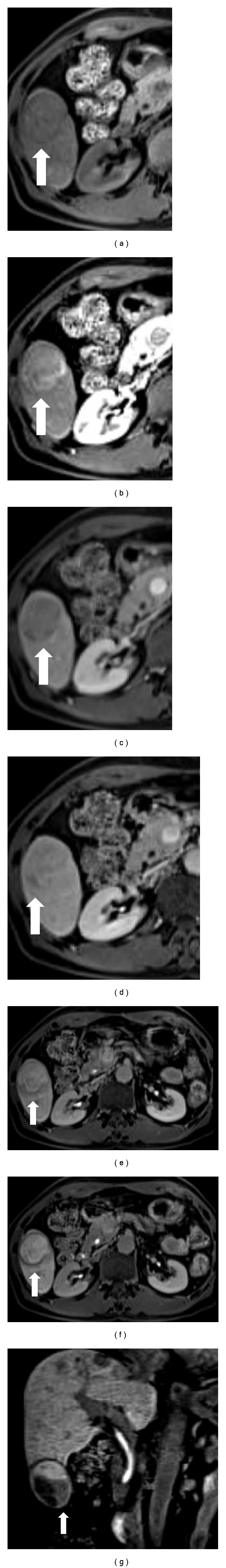
(a–d) Gadoxetate acid-enhanced MRI in the precontrast, arterial, venous, and equilibrium phases of Hepatitis B virus carrier showing a nodule in segment 6 of the liver with early arterial enhancement and late-phase washout compatible with HCC. (e), (f) Gadoxetate acid-enhanced MRI in the hepatobiliary phase 10–20 minutes after injection, showing progressively hyperintense portions of the nodule, implying lesional uptake, in a heterogeneous pattern. Note the hypointense pseudocapsule. Final surgical histopathology showed moderately differentiated Edmondson-Steiner grade II HCC. (g) Coronal view.

Figures 1(a)–1(c) illustrate the features of a typical HCC on gadoxetate acid-enhanced MRI. Figures 2(a)–2(d) demonstrate how gadoxetate acid-enhanced MRI can assist in the characterization of a non-specific, non-enhancing lesion on triphasic CT scan. Figures 3(a)–3(f) demonstrate another HCC with hepatobiliary excretion on gadoxetate acid-enhanced MRI.

### 4.1. Differentiating HCC from Regenerative or Dysplastic Nodules

Regenerative or dysplastic nodules are theoretically not malignant and hence may be expected to exhibit normal expression of the uptake transporter OATP1 and the excretory transporter MRP2. They take up contrast material and appear enhanced unlike most HCC [65]. Kudo reported that the differentiation of HCC from premalignant lesion can be achieved with 93% accuracy when investigated with gadoxetate acid-enhanced MRI [66]. However, as hepatocarcinogenesis is a stepwise continuum, a variable proportion of high-grade dysplastic nodules will begin to show lack of uptake of gadoxetate acid, resulting in overlap with early HCCs [67]. This highlights the potential pitfall in these borderline category cases. Currently, the Japan Liver Oncology Group (JLOG) is conducting a clinical trial to address this issue, to determine the frequency of dysplastic lesions appearing as hypointense, isointense, or hyperintense lesion in the hepatocyte phase [68]. Preliminary data from an Italian study suggests that a proportion of hypointense nodules on hepatocyte phase are high-grade dysplastic nodules and not always specific for HCCs [67]. From a practical standpoint, it may be appropriate to follow up these difficult nodules with interval imaging if they are smaller than 1.5 cm, whilst a more proactive approach such as biopsy may be advocated if lesions are larger than 1.5 cm since larger lesions tend to have a higher risk of malignancy or show microvascular invasion [69, 70].

### 4.2. Differentiating HCC from Hypervascular/Arterial Enhancing Pseudolesions

Arterioportal shunts are also one of the main mimickers of hypervascular HCCs on conventional dynamic contrast-enhanced CT and MRI [71, 72]. These are relatively of higher prevalence in the cirrhotic liver and appear as flash-enhancing lesions ranging from 5 to 20 mm and are typically not visible on other phases or sequences. However, as up to 50% of all flash-enhancing foci are eventually found to be HCCs, confident diagnosis at a single time-point is difficult without the benefit of serial followup. However, Motosugi and Sun et al. recently reported that gadoxetate acid-enhanced hepatocyte-phase MR images and diffusion weighted images are useful for distinguishing hypervascular pseudolesions from hypervascular HCCs [72, 73].

### 4.3. Differentiating HCC from Focal Nodular Hyperplasia (FNH)

Although regarded as the second most common benign tumor of the liver, FNH is less of a consideration in the cirrhotic liver. Nonetheless, they can be confidently distinguished from adenomas/metastases on gadoxetate acid-enhanced MRI as they typically appear as isointense or hyperintense on hepatocyte-phase images due to the presence of functioning hepatocytes and the presence of biliary canaliculi. Accurate characterization of FNH has been reported as high as 88% [74, 75]. Unnecessary biopsies, operations or close monitoring with 3–6 monthly MR or ultrasound imaging can be avoided.

**Figure 4:**
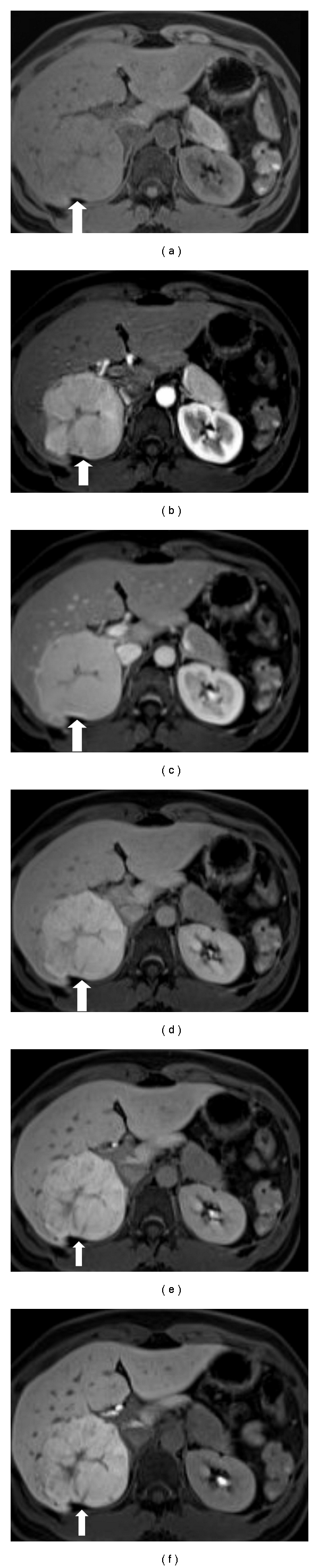
(a–d) Gadoxetate acid-enhanced MRI in the precontrast, arterial, venous, and equilibrium phases of 37-year-old female non-Hepatitis B or C virus carrier showing a large mass in the right lobe of the liver with early arterial enhancement and persistent late-phase enhancement with a small central hypointense scar. (e, f) Gadoxetate acid-enhanced MRI in the hepatobiliary phase 10–20 minutes after injection showing progressively hyperintense enhancement, in a homogeneous pattern apart from the hypointense small central scar. The MRI findings are typical for focal nodular hyperplasia.

 Figures 4(a)–4(f) demonstrate typical FNH features on gadoxetate acid-enhanced MRI.

### 4.4. Differentiating HCC from Liver Adenoma

Hepatic adenoma is a rare, benign liver tumor that predominantly affects women who take oral contraceptive pills. Like FNH, adenomas are typically hypervascular during the arterial phase but there is no central scar. In the hepatobiliary phase, it is thought that adenomas do not typically accumulate gadoxetate acid due to absence of functioning biliary elements unlike FNH. However, a few cases with hyperintense appearance in the hepatobiliary phase have been reported [76–78]. Currently, there is little published data to confirm the predominant pattern for adenomas, and larger studies with histopathological confirmation are needed.

## 5. Gadoxetate Acid: Sensitivity, Specificity, and Accuracy in HCC Detection in Comparison with Other Types of Contrast Agents or Imaging Techniques

Earlier studies comparing the diagnostic performance of gadoxetate acid-enhanced MRI against unenhanced MRI [75, 79, 80] and biphasic spiral CT [81, 82] showed clear superiority of gadoxetate acid-enhanced MRI over the other two in the detection and characterization of focal liver lesions, with as high as 10% increase in sensitivity [75, 79, 80] as compared to the unenhanced scan and 20% increase in sensitivity and 9% increase in specificity when compared to biphasic CT [81, 82]. This increase in diagnostic performance is notably significant for lesions smaller than 1 cm. At present, multidetector CT (MDCT) has surpassed spiral CT as the imaging of choice for the evaluation of focal liver lesion.

### 5.1. Evaluation against MDCT

In 2009, Kim et al. [59] reported his results on the diagnostic performance of gadoxetic acid-enhanced MRI and MDCT on the detection of HCC. His study population comprised of 83 HCCs (75 moderately-differentiated HCCs, 5 well-differentiated HCCs, 3 poorly-differentiated HCCs) with a mean size of 2.9 cm. Forty-eight percent of this population had Child-Pugh A cirrhosis; the rest had chronic hepatitis. The group found that although there is a trend for gadoxetate acid-enhanced MRI to have better performance in the detection of HCC, especially for those smaller or equal to 1 cm in size, there is otherwise no statistical significance in the performance of the two. The sensitivity was 91.6–94% in the gadoxetate group versus 82.2%–92.8% in the MDCT group. It is important to keep in mind that this study comprised mostly larger-sized tumors that are moderately-differentiated on the background of good liver function.

In the same year, another Korean group [83] published a statistically superior diagnostic accuracy result of HCC detection with gadoxetate acid-enhanced MRI when compared to MDCT. Here, 81 HCCs with a mean size of 1.5 cm were analysed by 2 observers. The group reported 91.4% sensitivity in the gadoxetate group versus 71.6% sensitivity in the MDCT group, with 24.7% higher percentage of HCC detection in smaller lesions (<1.5 cm). No nodules were missed at MRI but 4/81 nodules that were seen on MDCT were not verifiable on gadoxetate acid-enhanced MRI. It is important to note that more than 50% of the population had cirrhosis but not all had histological confirmation. 

Finally, in 2010, Martino et al. [84] also found that gadoxetate acid-enhanced MRI yielded superior diagnostic performance in HCC detection in the 87 HCCs (mean size 1.8 cm) on the background of liver cirrhosis, in both the diagnostic accuracy and sensitivity, when compared with those analysed by MDCT. Diagnostic accuracy was 88% and 74% and average sensitivity was 85% and 69% for the gadoxetate group and the MDCT group, respectively. This increased performance is clear for lesions smaller than 1.5 cm as well. However, it must be noted that only 61% of the population had histological diagnosis.

### 5.2. Evaluation against Other Contrast Agents

#### 5.2.1. Comparing Gadoxetate Acid-Enhanced MRI and Gadobenate Dimeglumine-Enhanced MRI in the Detection and Characterization of HCC

Although prior study showed that both gadoxetate acid and gadobenate dimeglumine can achieve similar enhancement in normal liver, this finding is different in the cirrhotic liver. Filippone [85] found, in his multicenter trial comprising of 70/295 patients with cirrhotic livers, that use of gadoxetate acid resulted in better liver enhancement in the overall (57.24% versus 32.77%) and in the cirrhotic subgroup (57.00% versus 26.85%) population than when gadobenate dimeglumine is used. The enhancement pattern of liver parenchyma for the cirrhotics on gadoxetate acid-enhanced MRI, however, was comparable to the enhancement ability achieved in the overall population using gadoxetate acid (57.00% versus 57.24%). 

Based on these above findings, one would think that this means definite improvement in HCC detection in gadoxetate acid-enhanced MRI compared to gadobenate dimeglumine-enhanced MRI in the detection of HCCs in the cirrhotic subgroup because of presumed increase in liver-to-lesion contrast. However, Park et al. [86]—who, to the authors' best knowledge, is the only group that compared the diagnostic performance of gadoxetate acid- and gadobenate dimeglumine-enhanced MRI for the detection of hepatocellular carcinoma—reported similar diagnostic performance of gadoxetic acid- and gadobenate-enhanced MRI. It is important to note here that the study population is small (18 patients with 22 HCCs), with a relatively large-sized HCCs (mean size of 2.9 cm) and in patients with good liver function. Overall, the authors still advocate the use of gadoxetate acid due to the other additional benefits of earlier enhancement and shorter total examination time.

#### 5.2.2. Comparing Gadoxetate Acid-Enhanced MRI and SPIO-Enhanced MRI in the Detection and Characterization of HCC

SPIO has been used and proven effective in the detection of malignant focal liver lesions, both HCC and metastases [87, 88], with a sensitivity range of 68%–97% [89, 90]. 

Kim et al. [91] reported significantly improved sensitivity (90.7% versus 84.7%) in the detection of 118 histologically confirmed HCCs by gadoxetate acid-enhanced MRI when compared with SPIO-enhanced study. The authors noticed that the improved sensitivity is most pronounced for lesions greater than 1.5 cm in size and that lesion characterization with certainty remains an issue with gadoxetate acid-enhanced MRI, despite its superior detection rate.

Lee et al. [92] reported similar diagnostic performance between gadoxetate acid- and ferucarbotran-enhanced MRI on a 3.0-T unit in a population of 38 histologically proven HCCs. However, it should be noted that the majority of the HCCs in the study were of relatively larger size (mean size of tumors is 2.8 cm), and 34/38 HCCs were moderately differentiated HCCs. 

Okada et al. [93] set out to compare the diagnostic performance between the two types of contrast-enhanced MRI in characterizing enhancement patterns of well-differentiated HCC and dysplastic nodules. They can have similar MRI features, making accurate radiological diagnosis difficult. His study population of HCCs was different from the study by Lee. In this prospective study analyzing 37 histologically proven HCC in 36 patients: 22/37 were well-differentiated HCCs with a mean size of 14 mm (sizes ranging from 6 to 28 mm; 15/37 were moderate to poorly-differentiated HCCs (as compared to the study by Lee JY where 35/38 were moderately-differentiated HCCs) with sizes ranging from 13–46 mm; 4 were dysplastic nodules with a mean of 16 mm (sizes ranging from 13 to 22 mm). Okada found gadoxetate acid-enhanced MRI to be more sensitive than ferucarbotran-enhanced MRI in the accurate evaluation of the enhancement patterns of his study population. However, one must note that 74% of patients in the study were Child-Pugh class A; Child-Pugh class C were excluded from the study.

## 6. Accepted Gadoxetate Acid-Enhanced MR Protocol

The current suggested protocol for gadoxetate acid-enhanced MR imaging of the liver comprises two main parts, as laid out below [69, 76]. In order to reduce the time the patient spends in the MRI room, the longer T2-weighted and diffusion-weighted sequences can be performed after the dynamic post-contrast phase, rather than prior to the injection of contrast as in conventional MRI protocols, without significant alteration of the lesional signal characteristics. The total scan time is slightly longer than conventional MR but the difference is minimized by this rearrangement of the sequences.

1. Precontrast sequences (similar to that of conventional MR imaging) includes the following.
  - Coronal single shot, fast spin echo T2-weighted sequences.
  - T1-weighted in/opposed phase. This combination sequence allows comparison of the varying signal intensities of the same lesion, further defining its true nature. This sequence is most helpful in the interpretation of fat-containing tissues or lesions, for example, in the determination of hepatic steatosis. Fatty lesions demonstrate “signal drop”—where fat, which is bright during the ‘in' phase, appears correspondingly darker in the “opposed” phase.
  - T1-precontrast sequence. This forms the baseline signal to which post-contrast images are compared to.
2. Administration of gadoxetate acid, either as a standard dose of 10 mls or 0.025 mmol/kg body weight of gadoxetate acid, given as an intravenous bolus at 1.5–2 ml/sec, flushed immediately with 20 mL saline.
3. Post-contrast sequences are then obtained in the following manner.
  - Dynamic imaging.
    - T1-weighted dynamic images are to be obtained immediately post-contrast administration. This includes the arterial, porto-venous, and equilibrium phase up to 5 minutes post-contrast images. These images evaluate a lesion's perfusion and washout characteristics.
  - Axial T2-weighted and diffusion-weighted sequences.
  - Hepatobiliary phase.
    - T1-weighted hepatobiliary phase in both axial and coronal views. These images are usually acquired at 10–20 minutes post contrast administration. This hepatobiliary phase utilizes the unique properties of gadoxetate acid, as discussed earlier, to yield additional valuable information for lesion characterization.

## 7. Area of Future Studies

Most HCCs arise in the background of cirrhosis. Most of these early small nodules (<2 cm) in the background of early liver cirrhosis have been shown to appear hypointense relative to the surrounding liver parenchyma on the hepatocyte-specific phase of gadoxetate acid-enhanced MRI [60, 61, 94–98], although the signal enhancement of cirrhotic liver parenchyma is not as strong as that of normal liver [69, 80]. However, challenges remain in three categories of patients: (1) those that have small lesions in the background of early liver cirrhosis—distinguishing the small HCCs from other premalignant nodules is difficult radiologically; (2) those with renal impairment—can gadoxetate acid be safely used in this group of patients?; (3) those with advanced or decompensated liver cirrhosis—suboptimal or no enhanced liver-to-lesion contrast can be achieved.

Cruite et al. [65] discussed the reasons behind the 3 unique diagnostic challenges faced in the diagnoses of HCCs in patients with advanced or decompensated cirrhosis. Firstly, there is expected impairment of contrast agent uptake due either to the reduced number of functional or the presence of dysfunctional hepatocytes. Secondly, there may be delayed or decreased biliary excretion from the impaired contrast uptake. Correspondingly, enhancement of the liver parenchyma, and the liver-to-lesion conspicuity, is decreased. In addition, there may also be pooling of contrast agent in the blood because of the significant reduction in the hepatic, and possibly renal, elimination as patients with advanced liver disease often have renal impairment as well, making gadoxetate acid behave like an ECF agent. Further studies are required to confirm the role of gadoxetate acid-enhanced MRI in the diagnosis of liver lesions in these groups of patients.

## 8. Summary

Gadoxetate acid-enhanced MRI of the liver has certain advantages over other imaging modalities in the detection and characterization of HCC in the high-risk liver. With increasing experience and application globally, it may potentially be established as the diagnostic imaging modality of choice in this setting.

## Figures and Tables

**Table 1:** Major differences between gadoxetate acid and gadobenate dimeglumine.

Properties in comparison	Gadoxetate acid	Gadobenate dimeglumine
% contrast uptake	50%	2–4%
Hepatobiliary phase image acquisition	10–45 minutes postcontrast administration	60–120 min postcontrast administration
Duration of liver enhancement	2 hrs	4 hrs
Clearance	50% biliary excretion, 50% renal excretion	2–4% biliary excretion, 96% renal excretion
Recommended dosage	0.025 mmol/kg, bolus injection at 2 mL/sec	0.1 mmol/kg bodyweight, bolus injection at 2 mL/sec

**Table 2:** 

Differences	SPIO	Hepatobiliary agents
Targeting cells	Kupffer cells	Functioning hepatocytes
Liver parenchyma	Black liver	White liver
Malignant liver lesion	White nodule	Black nodule
